# Genetic inactivation of the Fanconi anemia gene *FANCC *identified in the hepatocellular carcinoma cell line HuH-7 confers sensitivity towards DNA-interstrand crosslinking agents

**DOI:** 10.1186/1476-4598-9-127

**Published:** 2010-05-28

**Authors:** Andreas Palagyi, Kornelia Neveling, Ursula Plinninger, Andreas Ziesch, Bianca-Sabrina Targosz, Gerald U Denk, Stephanie Ochs, Antonia Rizzani, Daniel Meier, Wolfgang E Thasler, Helmut Hanenberg, Enrico N De Toni, Florian Bassermann, Claus Schäfer, Burkhard Göke, Detlev Schindler, Eike Gallmeier

**Affiliations:** 1 Department of Medicine II, Campus Grosshadern, Ludwig-Maximilians-University, Marchioninistrasse 15, 81377 Munich, Germany; 2 Department of Human Genetics, Biocenter, Julius-Maximilians-University, Am Hubland, 97074 Würzburg, Germany; 3 Department of Medicine III, Klinikum rechts der Isar, Technical University Munich, Ismaningerstrasse 22, 81675 Munich, Germany; 4 Department of Surgery, Campus Grosshadern, Ludwig-Maximilians-University, Marchioninistrasse 15, 81377 Munich, Germany; 5 Department of Pediatric Oncology, Hematology and Clinical Immunology, Heinrich Heine University, Moorenstrasse 5, 40225 Düsseldorf, Germany

## Abstract

**Background:**

Inactivation of the Fanconi anemia (FA) pathway through defects in one of 13 FA genes occurs at low frequency in various solid cancer entities among the general population. As FA pathway inactivation confers a distinct hypersensitivity towards DNA interstrand-crosslinking (ICL)-agents, FA defects represent rational targets for individualized therapeutic strategies. Except for pancreatic cancer, however, the prevalence of FA defects in gastrointestinal (GI) tumors has not yet been systematically explored.

**Results:**

A panel of GI cancer cell lines was screened for FA pathway inactivation applying FANCD2 monoubiquitination and FANCD2/RAD51 nuclear focus formation and a newly identified FA pathway-deficient cell line was functionally characterized. The hepatocellular carcinoma (HCC) line HuH-7 was defective in FANCD2 monoubiquitination and FANCD2 nuclear focus formation but proficient in RAD51 focus formation. Gene complementation studies revealed that this proximal FA pathway inactivation was attributable to defective FANCC function in HuH-7 cells. Accordingly, a homozygous inactivating *FANCC *nonsense mutation (c.553C > T, p.R185X) was identified in HuH-7, resulting in partial transcriptional skipping of exon 6 and leading to the classic cellular FA hypersensitivity phenotype; HuH-7 cells exhibited a strongly reduced proliferation rate and a pronounced G2 cell cycle arrest at distinctly lower concentrations of ICL-agents than a panel of non-isogenic, FA pathway-proficient HCC cell lines. Upon retroviral transduction of HuH-7 cells with *FANCC *cDNA, FA pathway functions were restored and ICL-hypersensitivity abrogated. Analyses of 18 surgical HCC specimens yielded no further examples for genetic or epigenetic inactivation of *FANCC*, *FANCF*, or *FANCG *in HCC, suggesting a low prevalence of proximal FA pathway inactivation in this tumor type.

**Conclusions:**

As the majority of HCC are chemoresistant, assessment of FA pathway function in HCC could identify small subpopulations of patients expected to predictably benefit from individualized treatment protocols using ICL-agents.

## Background

Fanconi anemia (FA) is a rare recessive disorder, characterized by congenital skeletal abnormalities, progressive bone marrow failure and an increased cancer susceptibility [1]. The disease is caused by bi-allelic mutations in one of 13 FA genes, all of which have now been identified [2]. The FA genes appear to act in a common pathway of DNA damage signalling and DNA remodelling, distal parts of which interact with regulators of cell cycle control and DNA repair, especially the repair of DNA interstrand-crosslinks.

There are three consecutive compartments of the FA pathway [3]. The proximal compartment consists of eight FA proteins (A, B, C, E, F, G, L, and M), which form a nuclear FA core complex upon activation. This complex functions as an E3 ligase and mediates the monoubiquitination of FANCD2 [4], which represents the central FA pathway protein. FANCI also becomes monoubiquitinated during this process [5,6] and cooperates with FANCD2 in the ID (FANCI/FANCD2) complex [7,8]. The activated proteins of the ID complex subsequently co-localize with proteins of the distal FA pathway compartment (FANCD1/BRCA2, FANCN, and FANCJ) and with other DNA-repair proteins such as RAD51 at sites of DNA-damage. Cells having a defect in one of the proximal FA core complex genes are deficient in FANCD2/FANCI monoubiquitination and FANCD2/FANCI nuclear focus formation. Similarly, cells having a defect in one of the distal FA pathway genes *FANCD1 *or *FANCN *are deficient in RAD51 focus formation [8-12]. Cells with a defect in one of the ID complex proteins lack the respective protein and are defective in monoubiquitination of the other. Thus, inactivation of the FA pathway can comprehensively be identified at the cellular level by assays detecting FANCD2 monoubiquitination and FANCD2/RAD51 focus formation.

FA pathway inactivation occurs sporadically in a variety of tumor types of non-FA patients, suggesting a role of the FA genes in tumor suppression or maintenance of genomic stability among the general population. Distal FA pathway inactivation via mutations in *FANCD1 *occurs in familial cases of breast (2-25%) [13] and ovarian cancer (2-6%) [14], in familial cases of pancreatic cancer (17%) [15] and in sporadic cancers of various tumor entities [16-18]. In comparison, genetic inactivation of the proximal FA pathway appears to occur infrequently in tumors among the general population and has, in terms of GI cancers, yet only been reported in pancreatic cancer, where it was associated with rare mutations in *FANCC *or *FANCG *[19,20]. In addition, germline mutations of *FANCC *might contribute to the tumorigenesis or tumor progression of pancreatic cancer [20-22]. Finally, epigenetic inactivation of the proximal FA pathway via hypermethylation of *FANCF *has been reported in a variety of tumor entities [23-27], but its significance is not yet well understood [28,29].

Unlike the setting in FA patients, FA pathway-deficient tumors arising in patients of the general population harbor the FA gene defect exclusively in the tumor cells, whereas stroma and all other non-malignant cells lack the defect, thus representing a tumor-specific, absolute biochemical difference [30]. As FA pathway-deficient cells are hypersensitive to ICL-agents and PARP inhibitors, FA pathway inactivation in tumors represents a promising target for rational, genotype-based anticancer therapy [31-38].

The prevalence of FA pathway defects has not yet been systematically investigated in GI cancer. We assessed proximal and distal FA pathway function in 48 cell lines derived from gastric, pancreatic, colorectal, hepatocellular and cholangiocellular carcinomas applying assays for FANCD2 monoubiquitination and FANCD2/RAD51 focus formation [39]. We newly identified a single cell line, HuH-7, derived from a hepatocellular carcinoma (HCC), which exhibited a proximal FA pathway defect, ascribable to genetic *FANCC *inactivation. When compared to four other HCC cell lines, HuH-7 cells exhibited an increased sensitivity towards ICL-agents, which was reversible in these cells by genetic correction through *FANCC *overexpression. Our data represent the first evidence for genetic inactivation of the proximal FA pathway in HCC and further support the assumption that genetic inactivation of the proximal FA pathway is a rare event in solid tumors among the general population.

## Results

### GI cancer cell line-screening identifies proximal FA pathway-deficiency in HuH-7 cells

**Figure 1:**
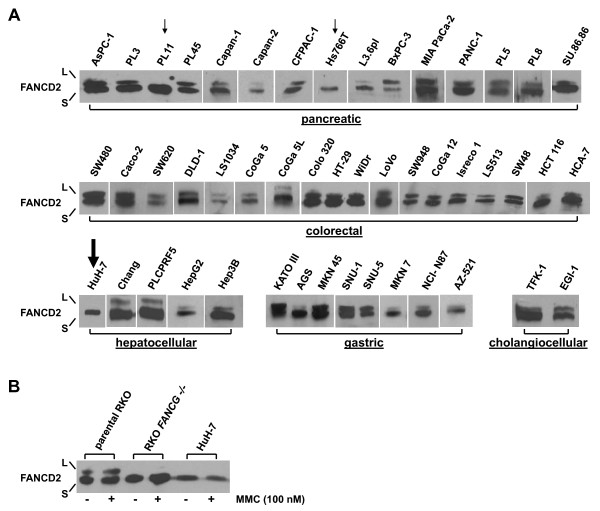
**Detection of monoubiquitinated (L) and non-monoubiquitinated (S) FANCD2 protein to assess proximal FA pathway status in GI cell lines**. **(A) **Immunoblotting to assess FANCD2 monoubiquitination in 48 tumor cell lines of pancreatic, colorectal, hepatocellular, gastric and cholangiocellular origin. Pancreatic cell lines were described previously, including the FA pathway-deficient cell lines Hs766T and PL11 (*small arrows*), and served as controls. The HCC cell line HuH-7 (*big arrow*) lacked spontaneous FANCD2 monoubiquitination. **(B) **FANCD2 immunoblotting of RKO cells (positive control), derived RKO *FANCG -/- *cells (negative control) and HuH-7 cells upon treatment with MMC at 100 nM for 24 h to stimulate potential previously undetectable FANCD2 monoubiquitination.

**Figure 2:**
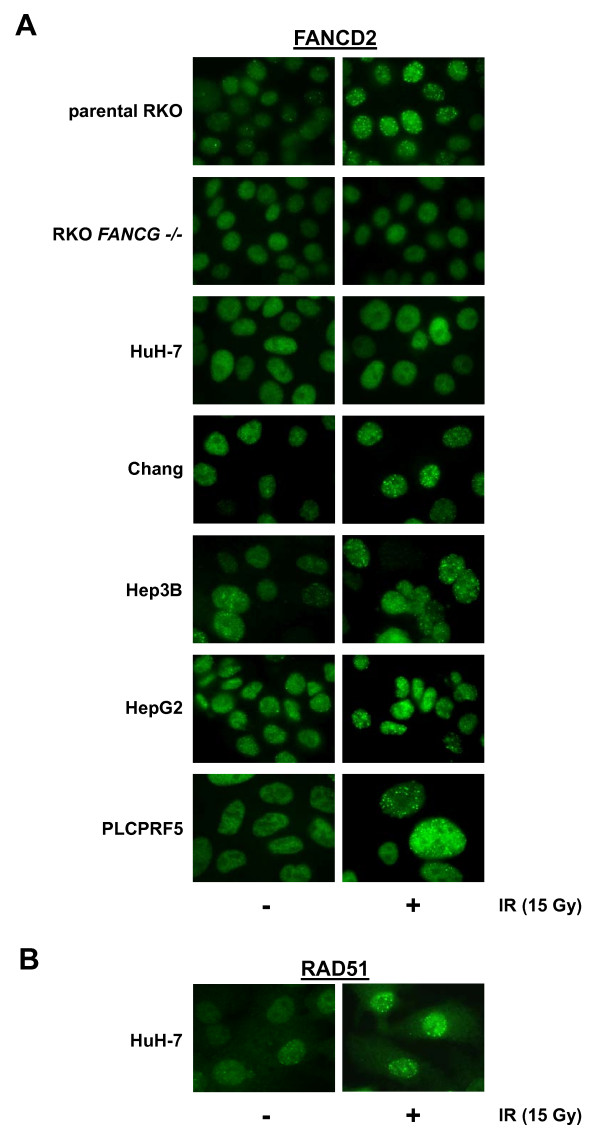
**FANCD2 and RAD51 nuclear focus formation to assess proximal and distal FA pathway function**. **(A) **FANCD2 nuclear focus formation in RKO cells (positive control), derived RKO *FANCG *-/- cells (negative control), and a panel of five HCC cell lines including HuH-7, either treated with IR at 15 Gy for 6 h or left untreated. HuH-7 and RKO *FANCG *-/- cells lacked FANCD2 focus formation. **(B) **RAD51 nuclear focus formation in HuH-7 cells either treated with IR at 15 Gy for 6 h or left untreated.

48 gastrointestinal cancer cell lines (18 colorectal, 15 pancreatic, 8 gastric, 5 hepatocellular and 2 cholangiocellular carcinomas) were screened for proximal and distal FA pathway inactivation using FANCD2 immunoblotting, FANCD2 and RAD51 nuclear focus formation (Fig. 1A, Table 1). Results on FA pathway inactivation in pancreatic cancer cell lines have been previously reported and were confirmed, including defective FANCD2 monoubiquitination due to inactivating mutations in *FANCG *and *FANCC *in Hs766T and PL11 [20,22], respectively, and defective RAD51 focus formation due to an inactivating *FANCD1 *mutation in Capan-1 [17]. Among all samples newly tested in the present study, we identified one cell line without detectable FANCD2 monoubiquitination, the HCC line HuH-7 (Fig. 1A). To stimulate previously undetectable FANCD2 monoubiquitination, HuH-7 cultures were pre-treated with mitomycin C (MMC) at 100 nM for 24 h. Despite such challenge, HuH-7 did not display any trace of monoubiquitinated FANCD2. RKO cells and derived clones harboring an engineered disruption of *FANCG *or *FANCC *were used as controls [36] (Fig. 1B). Likewise, no FANCD2 focus formation was observed in HuH-7 after treatment with irradiation (IR) at 15 Gy 6 h prior to workup (Fig. 2A). In contrast, RAD51 focus formation was readily detectable (Fig. 2B), confirming a proximal FA pathway defect, whereas distal FA/BRCA pathway function remained undisturbed.

**Table 1:** FANCD2- and RAD51-nuclear focus formation in 48 GI cancer cell lines

Cell line	Cancer type	FANCD2	RAD51	Cell line	Cancer type	FANCD2	RAD51
AGS	gastric	+	+	L3.6pl	pancreatic	+	+
AsPC-1	pancreatic	+	+	LoVo	colorectal	+	+
AZ-521	gastric	+	+	LS1034	colorectal	+	+
BxPC-3	pancreatic	+	+	LS513	colorectal	+	+
Caco-2	colorectal	+	+	MIA PaCa-2	pancreatic	+	+
**Capan-1**	**pancreatic**	**+**	**-**	MKN 45	gastric	+	+
Capan-2	pancreatic	+	+	MKN 7	gastric	+	+
CFPAC-1	pancreatic	+	+	NCI-N87	gastric	+	+
Chang	hepatocellular	+	+	PANC-1	pancreatic	+	+
CoGa 12	colorectal	+	+	**PL11**	**pancreatic**	**-**	**+**
CoGa 5	colorectal	+	+	PL3	pancreatic	+	+
CoGa 5L	colorectal	+	+	PL45	pancreatic	+	+
Colo 320	colorectal	+	+	PL5	pancreatic	+	+
DLD-1	colorectal	+	+	PL8	pancreatic	+	+
EGI-1	cholangiocellular	+	+	PLCPRF5	hepatocellular	+	+
HCA-7	colorectal	+	+	SNU-1	gastric	+	+
HCT 116	colorectal	+	+	SNU-5	gastric	+	+
Hep3B	hepatocellular	+	+	SU.86.86	pancreatic	+	+
HepG2	hepatocellular	+	+	SW48	colorectal	+	+
**Hs766T**	**pancreatic**	**-**	**+**	SW480	colorectal	+	+
HT-29	colorectal	+	+	SW620	colorectal	+	+
**HuH-7**	**hepatocellular**	**-**	**+**	SW948	colorectal	+	+
Isreco 1	colorectal	+	+	TFK-1	cholangiocellular	+	+
KATO III	gastric	+	+	WiDr	colorectal	+	+

(+) detectable or (-) no detectable nuclear focus formation of FANCD2 or RAD51, respectively, in the indicated cell lines, 6 h after treatment with IR at 15 Gy. FA pathway-deficient cell lines are depicted in bold.

### Gene complementation studies reveal defective FANCC function in HuH-7 cells

**Figure 3:**
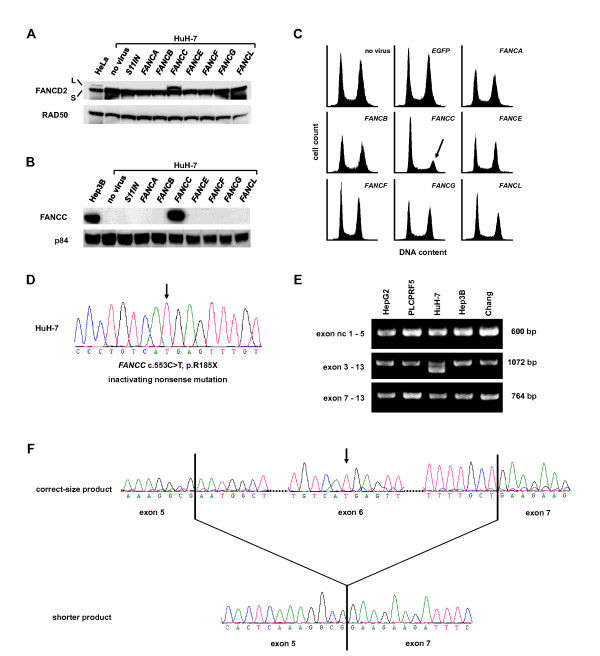
**Gene complementation studies identifying an inactivating *FANCC *nonsense mutation in HuH-7**. **(A) **FANCD2 monoubiquitination in FA pathway-proficient control HeLa cells and HuH-7 cells transduced with the indicated FA or control cDNA constructs. All cultures were exposed to MMC at 150 nM for 15 h. RAD50 served as loading control. Only transduction with *FANCC *restored FANCD2 monoubiquitination in HuH-7 cells. **(B) **FANCC immunoblotting using the indicated cell lysates. The nuclear protein p84 served as loading control. Only HuH-7 cells transduced with *FANCC *and FA pathway-proficient Hep3B control cells displayed FANCC protein. **(C) **Cell cycle distributions of HuH-7 cells transduced with the indicated FA or control cDNA constructs. All cultures were exposed to MMC at 33 nM for 15 h. Only transduction with *FANCC *abrogated the pronounced G2 cell cycle arrest upon MMC in HuH-7 cells. **(D) **Chromatogram of the homo- or hemizygous *FANCC *mutation (*arrow*) identified in HuH-7 cells. **(E) **RT-PCR using three different primer pairs amplifying a region from *FANCC *non-coding exon 1 to coding exon 5, or from coding exons 3 to 13, or from coding exons 7 to 13, respectively, using cDNA from five HCC cell lines. Only HuH-7 cells exhibited aberrant mRNA splicing lacking exon 6. **(F) **Direct sequencing of the two distinct RT-PCR products confirmed that the product having the correct size represented the reference *FANCC *sequence except for the c.553C > T mutation, whereas the shorter product lacked exon 6, exhibiting a direct exon 5/7 junction.

To identify the FA gene responsible for proximal FA pathway inactivation, HuH-7 cells were transduced with retroviral constructs containing full-length cDNAs of *FANCA*, *FANCB*, *FANCC*, *FANCE*, *FANCF*, *FANCG *or *FANCL*. FANCD2 monoubiquitination was assessed following exposure of the transduced cultures to MMC. Restoration of FANCD2 monoubiquitination was observed only after complementation of HuH-7 cells with the *FANCC *gene construct, whereas all other constructs had no discernible effect (Fig. 3A). Consequently, exogenous FANCC protein expression was confirmed in *FANCC*-transduced HuH-7 cultures (Fig. 3B). To validate functional FA pathway restoration upon transduction of *FANCC*-cDNA, the cell cycle profiles of the transduced cells were analyzed 48 h after treatment with MMC. The pronounced G2 arrest, observed in non-*FANCC*-complemented HuH-7 cells was restored to normal specifically after transduction with *FANCC*, but not after transduction with any of the other FA or control constructs (Fig. 3C).

### HuH-7 cells harbor a homozygous inactivating nonsense FANCC mutation in exon 6

Direct genomic sequencing of the coding exons and adjacent intron portions of *FANCC *in HuH-7 revealed a homo- or hemizygous inactivating nonsense mutation c.553C > T, p.R185X in coding exon 6 (Fig. 3D). No other sequence changes were identified.

### Aberrant splicing leads to a truncated FANCC mRNA lacking exon 6 in HuH-7 cells

Using reverse-transcription PCR (RT-PCR) with primers flanking a region that includes exon 6, we identified an additional truncated *FANCC *gene transcript in HuH-7 cells, which was not detectable in four HCC control cell lines Chang, Hep3B, HepG2 and PLCPRF5 (Fig. 3E). Upon gel-separation of the two RT-PCR products amplified from HuH-7 cDNA, direct sequencing confirmed that the correctly sized band represented the reference *FANCC *sequence except for the described c.553C > T nonsense mutation, while the smaller band represented the reference *FANCC *sequence lacking exon 6, leading to a direct exon 5/7 junction (Fig. 3F).

### HuH-7 cells are hypersensitive towards ICL-agents as compared to other HCC lines

**Figure 4:**
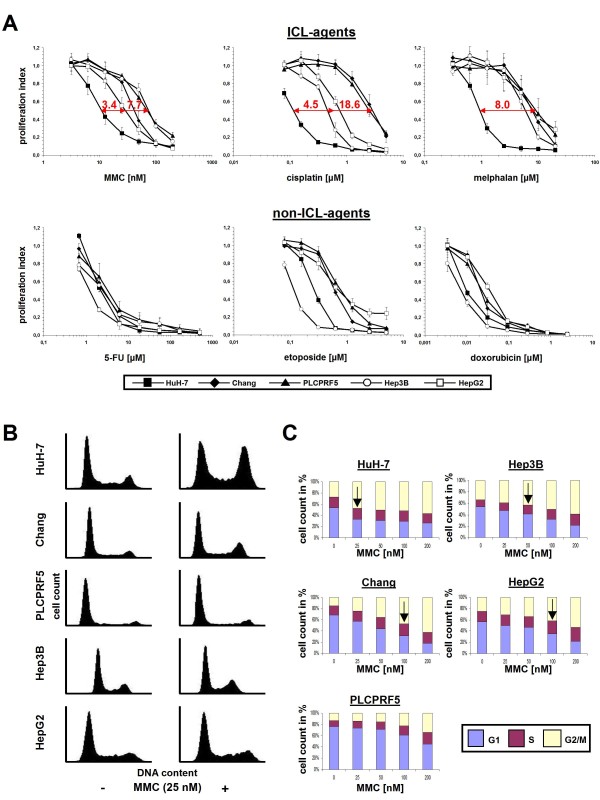
**Hypersensitivity of HuH-7 cells specifically towards ICL-agents**. **(A) **Cell proliferation assays to compare the sensitivity of five HCC cell lines towards ICL- and non-ICL-chemotherapeutic agents. HuH-7 cells were hypersensitive towards treatment with any of three ICL-agents as compared to four FA pathway-proficient HCC lines (upper panel: MMC, cisplatin, melphalan), with IC50 ratios ranging from 3.4- to 18.6-fold (*arrows*, indicated in red), but not towards any of three non-ICL-agents (lower panel: 5-FU, doxorubicin, etoposide). Error bars represent SEM of three independent experiments. **(B) **Flow cytometric cell cycle profiles of five HCC cell lines 48 h after treatment with MMC at the indicated concentrations. A pronounced G2 arrest (defined here as a cell fraction of >40% in G2 [36]) at low-dose MMC (25 nM) was observed only in HuH-7 cells. **(C) **Graphic representation of the cell cycle distributions from a representative experiment displaying the fraction of cells in G1, S or G2 at the indicated MMC concentrations for the indicated HCC cell lines. *Arrows *indicate the lowest dose at which a G2 arrest of more than 40% of the cells was observed.

Five HCC cell lines (Chang, Hep3B, HepG2, HuH-7 and PLCPRF5) were treated either with common ICL-agents (MMC, cisplatin and melphalan) or with common non-ICL chemotherapeutics (5-fluorouracil (5-FU), etoposide and doxorubicin). The sensitivity towards ICL-agents was clearly dissimilar between the FA pathway-proficient cell line panel and *FANCC*-deficient HuH-7 cells, with the latter displaying a distinct hypersensitivity (inhibitory concentration 50% (IC50) ratios [40] ranging between 3.4 to 18.6 as compared to the other HCC lines) (Fig. 4A, upper panel). In contrast, treatment with non-ICL chemotherapeutics did not distinguish HuH-7 as being most sensitive (Fig. 4A, lower panel). The most consistent difference in ICL-agent sensitivity between the FA-pathway proficient HCC cell lines and *FANCC*-deficient HuH-7 cells was observed using melphalan. This agent has previously been described to have the strongest effect among 8 ICL-agents tested in an isogenic FA cancer cell model [36]. Cell cycle distributions of the five HCC cell lines, analyzed upon treatment with MMC for 48 h, revealed a pronounced G2 arrest (defined here by a fraction of >40% of cells in G2) [36] at low MMC concentrations (25 nM) only in HuH-7 cells (Fig. 4B), whereas a similar G2 arrest was achieved in a dose-dependent manner in all HCC cell lines except PLCPRF5 at higher MMC concentrations (50 to 100 nM) (Fig. 4C).

### ICL-hypersensitivity of HuH-7 cells is abrogated upon exogenous FANCC expression

**Figure 5:**
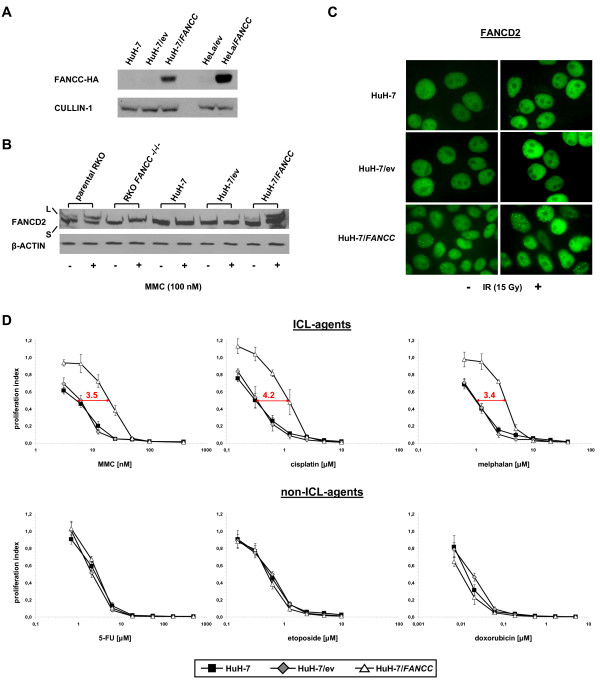
**Abrogation of the ICL-hypersensitivity phenotype in HuH-7 through exogenous *FANCC *expression**. **(A) **Immunoblotting for the detection of FANCC-HA in parental HuH-7 cells (HuH-7) and HuH-7-derived clones retrovirally transduced with either empty vector-pMSCV (HuH-7/ev) or with pMSCV-*FANCC *(HuH-7/*FANCC*). HeLa cells transduced with pMSCV-*FANCC *were used as controls. CULLIN-1 served as loading control. FANCC-HA was detectable only in HuH-7/*FANCC *and control HeLa/*FANCC *cells. **(B) **FANCD2 monoubiquitination in parental HuH-7, HuH-7/ev and HuH-7/*FANCC *cells, respectively, either treated with MMC at 100 nM for 24 h or left untreated. RKO cells and derived RKO *FANCC-/-/- *cells were used as controls. β-ACTIN served as loading control. Monoubiquitinated FANCD2 was detectable only in HuH-7/*FANCC *and parental RKO control cells and was inducible upon MMC-treatment, confirming correction of proximal FA pathway function through exogenous *FANCC *expression in HuH-7. **(C) **FANCD2 nuclear focus formation in parental HuH-7, HuH-7/ev and HuH-7/*FANCC *cells either treated with IR at 15 Gy for 6 h or left untreated. FANCD2 focus formation was detectable only in HuH-7/*FANCC *cells. **(D) **Cell proliferation assays to compare the sensitivity of parental HuH-7, HuH-7/ev and HuH-7/*FANCC *cells towards ICL- and non-ICL-chemotherapeutic agents. HuH-7/*FANCC *cells were less sensitive towards treatment with ICL-agents than parental and HuH-7/ev control cells (upper panel: MMC, cisplatin, melphalan), with IC50 ratios ranging from 3.4- to 4.2-fold (*arrows*, indicated in red), whereas sensitivity towards non-ICL-agents (lower panel: 5-FU, doxorubicin, etoposide) remained virtually unchanged. Error bars represent SEM of three independent experiments.

HuH-7 cells were stably transduced using retroviral constructs containing either full length *FANCC*-cDNA (HuH-7/*FANCC*), tagged with recombinant influenza virus hemagglutinin (HA-tag), or empty vector (HuH-7/ev). Consecutively, exogenous *FANCC *expression was confirmed (Fig. 5A). HuH-7/*FANCC *cells, but not parental HuH-7 or HuH-7/ev control cells, displayed restored proximal FA-pathway function as demonstrated by constitutive and MMC-inducible [41] FANCD2 monoubiquitination (Fig. 5B) as well as proficiency in FANCD2 nuclear focus formation (Fig. 5C). Most importantly, sensitivity towards ICL-agents was significantly reduced in HuH-7/*FANCC *cells as compared to parental HuH-7 and HuH-7/ev control cells (Fig. 5D, upper panel), whereas sensitivity towards non-ICL-chemotherapeutics remained virtually unchanged (Fig. 5D, lower panel).

### No evidence for inactivation of FANCC, FANCG or FANCF in 18 HCC tissue specimens

**Figure 6:**
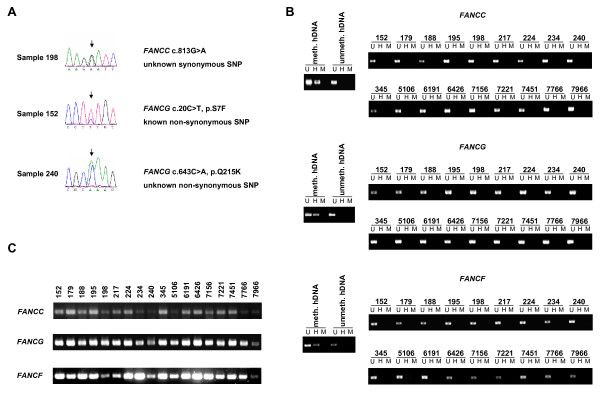
**Screening of 18 HCC tissues for inactivation of *FANCC, FANCF or FANCG***. **(A) **Chromatograms displaying the identified heterozygous sequence variants (*arrows*) in *FANCC *and *FANCG*. **(B) **PCR-based HpaII-restriction assays to determine methylation status of *FANCC*, *FANCF *and *FANCG *in 18 HCC tissues. The corresponding CpG island-containing regions were PCR-amplified before and after digestion using the indicated enzymes: U = undigested, H = HpaII digestion, M = MspI digestion. Universal methylated and unmethylated human DNA (hDNA) was used as controls. No hypermethylation of *FANCC*, *FANCF *or *FANCG *was detected in any of the 18 HCC samples. **(C) **RT-PCR to assess mRNA-expression of *FANCC*, *FANCF *and *FANCG *in 18 HCC tissues. All samples expressed *FANCC-*, *FANCF- *and *FANCG-*mRNA.

To assess the prevalence of proximal FA pathway inactivation in HCC, 18 surgical HCC tissue specimens were analyzed for inactivation of *FANCC, FANCF *or *FANCG *through screening for genetic mutations, epigenetic silencing through CpG hypermethylation or lack of mRNA-expression. On the genetic level, direct sequencing of the complete coding sequences of *FANCC*, *FANCF *and *FANCG *in 18 HCC samples identified a single not previously reported, heterozygous, synonymous *FANCC *sequence variant (c.813G > A) and two heterozygous, non-synonymous *FANCG *variants (c.20C > T, p.S7F; c.643C > A, p.Q215K) (Fig. 6A). No other sequence variants, especially no inactivating point mutations, small deletions, insertions, large intragenic deletions or complete homozygous gene deletions, were found. On the epigenetic level, HpaII-restriction assays yielded no evidence for *FANCC-*, *FANCF- *or *FANCG-*silencing through CpG hypermethylation (Fig. 6B) and consistently, *FANCC-*, *FANCF- *and *FANCG*-mRNA was detectable in all 18 HCC samples (Fig. 6C).

## Discussion

We report here the identification and functional characterization of an inactivating nonsense *FANCC *mutation in the HCC cell line HuH-7. This cell line was established from a well-differentiated HCC of a 57 year-old Japanese male patient [42], displays an aneuploid phenotype with an average number of 60 chromosomes, and is negative for HBV and HCV [43,44]. To our knowledge, this is the first evidence of genetic inactivation of the proximal FA pathway in a GI tumor entity other than pancreatic cancer.

The identified *FANCC *nonsense mutation c.553C > T, p.R185X in HuH-7 represents a known FA mutation, first described by Gibson et al. [45]. Interestingly, non-splice site nonsense mutations can cause exon-skipping through aberrant splicing [46], and accordingly, the c.553C > T mutation has been reported to cause partial transcriptional skipping of exon 6 of *FANCC *in an FA patient [45], a mechanism confirmed for HuH-7 in our study.

Unfortunately, no matched non-malignant tissue is available for HuH-7, precluding definitive genomic copy number analyses in regard to whether the identified *FANCC *mutation represents a homo- or hemizygous mutation in this cell line. However, according to copy number analyses by the Sanger Institute (Cambridge, UK) using high-density single nucleotide polymorphism (SNP) arrays (SNP 6.0) [47], HuH-7 harbors three nearly identical copies of the chromosomal arm 9q, where *FANCC *is located at 9q22.3, as evidenced by virtually exclusive homozygosity of all SNPs assessed on 9q. According to proposed evaluation models for the identification of LOH events where no matching normal tissue is available, these data are strongly suggestive (although not definitely evidentiary) of allelic loss of one copy of chromosome 9q including the non-mutated *FANCC *allele in the original tumor (or its precursor cells), followed by repeated duplication of the remaining chromosome 9q, including the mutated *FANCC *allele, later on [48-50]. Typical recurrent numerical chromosomal aberrations in HCC include losses on 1p, 4q, 8p, 13q, 16q, and 17p and gains on 1q, 8q, 16p and 20q [51]. Although chromosome 9 is rarely clonally altered on the cytogenetic level in HCC, LOH has been reported for several regions on chromosome 9 including the loci of the *FANCC *(9q22.3) and the *FANCG *(9p13) genes [52].

FA pathway defects in tumors require bi-allelic inactivation of one of at least 13 FA genes. On the one hand, these bi-allelic mutations could both be inherited, as applies to tumors occurring in FA-patients. On the other hand, mono-allelic germline mutations of distal FA pathway genes, such as *FANCD1*, *FANCN *or *FANCJ*, confer low to medium penetrance susceptibility for breast/ovarian cancer [12,13,53] and, as applies to *FANCD1 *and *FANCN*, also for pancreatic cancer [15,54-57]. In addition, inherited mono-allelic mutations of proximal FA pathway genes have been associated with the predisposition for or the accelerated development of certain tumors [21,54,55,58]. In particular, germline mutations of *FANCC *have been described in pancreatic cancer, associated with LOH in the tumor [21,22]. However, germline and somatic changes in *FANCC *and *FANCG *may have comparatively low penetrance for pancreatic cancer [55], which is supported by studies investigating germline mutations of upstream FA pathway genes in sporadic, yet FA-typical tumors among the general population [59]. Nevertheless, as the *FANCC *mutation in HuH-7 reported in our study represents an established FA mutation and was therefore most likely present in the germline of the patient in mono-allelic form, our data might indicate an increased risk for the development of HCC in individuals of the general population harboring this or other *FANCC *mutations.

The occurrence of an FA-associated *FANCC *mutation in HCC could also denote a tissue-specific susceptibility for the development of HCC in FA patients; The majority of solid malignancies seen in FA patients consists of head and neck or gynaecologic carcinomas (5.3%), as reported in a large meta-analysis of 1300 cases [60], but also 2.8% of all FA patients developed liver tumors. These tumors manifested at a significantly younger age than other solid malignancies (median age: 13 years for liver tumors as compared to 26 years for other solid malignancies). In fact, the cumulative probability of liver tumors in FA patients has been estimated to be 46% by age 50 [60]. However, the significance of these data regarding a potential liver-specific cancer susceptibility of FA patients is complicated by the observation that many liver tumors do not proceed to malignancy during the life span of FA patients [60,61]. In addition, there appears to be a strong association between androgen therapy, which is frequently used for the treatment of bone marrow failure in FA, and the occurrence of liver tumors [60-63]. Nevertheless, HCC represented the majority (58%) of tumors of 36 FA patients with androgen-related liver tumors in one study [61]. Thus, the association of FA with HCC could be attributable both to the primary tumorigenic effects of FA pathway inactivation in hepatocytes, as well as to potential secondary, amplifying or accelerating effects of androgen therapy in FA patients.

We demonstrated that HCC cells having an inactivated FA pathway display the classic cellular FA phenotype, including a specific hypersensitivity towards ICL-agents, illustrated in HuH-7 by a pronounced cell cycle arrest in G2 upon treatment with MMC at low concentrations and a strongly decreased proliferation rate as compared to a panel of non-isogenic HCC lines. Importantly, this ICL-hypersensitivity phenotype was reversed using an isogenic HuH-7 model of exogenous *FANCC *expression, confirming that ICL-hypersensitivity in these cells was attributable specifically to inactivation of *FANCC*. Of note however, IC50-ratios between *FANCC*-deficient and *FANCC*-proficient cells were partly smaller in the isogenic model than could have been expected from our results using the non-isogenic model. This observation could be attributable to FA pathway-independent ICL-sensitivity differences among the non-isogenic HCC cell lines, but could also provide further support for our previous hypothesis that constitutive exogenous *FANCC *over-expression does not completely substitute for physiologically regulated, endogenous *FANCC *expression [37,38].

It is well established that systemic chemotherapy lacks effectiveness in unselected HCC patients [64,65] and HCC are therefore considered largely chemoresistant, at least partially explaining the poor prognosis of this tumor entity [66]. Accordingly, guidelines are currently lacking also regarding the choice of chemotherapeutic agent to use in transarterial chemoembolization (TACE), one of the major treatment modalities for non-surgical patients at advanced HCC stages [67]. Our data indicate that non-FA patients having a FA-deficient HCC might predictably benefit from treatment using ICL-agents. Consequently, assessment of FA pathway function in HCC could facilitate individualized therapeutic approaches, using genotype-based patient stratification in regard to both systemic chemotherapy and TACE.

To get an estimate of the prevalence of *FANCC *inactivation in HCC, we sequenced cDNA derived from 18 surgical HCC tissue specimens to screen for genetic *FANCC *inactivation. We further screened these samples for hypermethylation of the *FANCC *promoter region and for lack of *FANCC *mRNA expression, as epigenetic *FANCC *inactivation has previously been reported in acute leukaemia and breast cancer [68,69]. Additionally, we included *FANCG *and *FANCF *in these analyses, as *FANCG *represents another proximal FA gene that has been described to be mutated in GI cancer, specifically in pancreatic cancer [20,22], while *FANCF *has been reported to be epigenetically inactivated in various tumor types [23-25,27]. On the genetic level, we found no further inactivating alterations, especially no evidence for complete homozygous gene deletions, inactivating point mutations, small deletions or insertions, in *FANCC*, *FANCG *or *FANCF*, respectively. The detected *FANCG *variant c.20C > T, p.S7F has been reported in an FA patient of the complementation group G in addition to pathogenic *FANCG *mutations [70]. There is no information available on the nature of the c.643C > A, p.Q215K variant in *FANCG*. However, LOH or a second sequence variant was not detected in that tumor either. Additionally, since only two to three overlapping PCR reactions were used to amplify the complete coding sequences of *FANCC*, *FANCG *and *FANCF*, respectively, most potential large intragenic deletions would have been detected. However, this mechanism of proximal FA gene inactivation occurs almost exclusively in *FANCA*, whereas it appears to be extremely rare in *FANCC *and has not at all been described in *FANCG *[20,71-73]. On the epigenetic level, we found no evidence for hypermethylation of *FANCC*, *FANCG *or *FANCF *in any of the 18 samples. Consistently, *FANCC*, *FANCG *and *FANCF *were expressed in all samples on the mRNA level.

Our negative screening results for proximal FA pathway inactivation in HCC were not unexpected, as a hypothetical high prevalence should have become evident earlier during clinical trials - manifesting as a selective chemosensitivity of the majority of HCC towards ICL-agents. Nevertheless, the lack of FA mutations in 18 HCC does not rule out rare occurrences of proximal FA pathway inactivation in HCC and is consistent with previous reports on the low prevalence of proximal FA pathway inactivation in various tumor entities among the general population [20-22,26,29,68]. Future studies applying a higher sample number are required to definitely determine the prevalence of FA pathway inactivation in HCC.

## Conclusions

In summary, we identified a HCC cell line harboring an inactivating mutation of the *FANCC *gene, specifically causing proximal FA pathway inactivation and the classic cellular ICL-hypersensitivity phenotype. Assessment of FA pathway function in HCC could thus represent a novel approach to identify small subgroups of HCC patients expected to predictably benefit from individualized treatment protocols using ICL-agents. However, as proximal FA pathway inactivation occurs at low frequency in HCC and other tumors among the general population, the development of rapid, economic and clinically applicable screening tests for proximal FA pathway inactivation, particularly in primary tumor tissues, remains an indispensable requirement to facilitate future clinical studies [38].

## Materials and methods

### Cell lines, culture conditions and HCC tissue samples

Cell lines PL3, PL5, PL8, PL11 and PL45 were kindly provided by S.E. Kern (Johns Hopkins University, Baltimore, Maryland). Cell lines AZ-521, CoGa-5, CoGa-5L, Coga-12, HCA-7, Isreco 1, MKN7, MKN45, NCl-N87 and L3.6pl were kindly donated by C.J. Bruns, M. Gerhard, F.T. Kolligs and M. Ogris (Technical University and Ludwig-Maximilians-University, Munich, Germany). The remaining cell lines were purchased from the European Collection of Cell Cultures (Sigma-Aldrich, Munich, Germany) or the American Type Culture Collection (LGC Standards, Wesel, Germany). RKO cells harboring an engineered disruption of the *FANCG *or *FANCC *gene have been described [36]. Cells were grown in DMEM supplemented with 10% fetal calf serum, L-glutamine and penicillin/streptomycin (PAA, Cölbe, Germany). The 18 HCC tissue samples were a kind gift of the Human Tissue and Cell Research-Trust (HTCR, Regensburg, Germany) and originated from HCCs that where surgically removed at the Ludwig-Maximilians-University (Munich, Germany).

### Immunoblotting

Cells were treated with MMC (Sigma) at 100 nM for 24 h or left untreated. Consecutively, cells were lysed and protein extracts boiled and loaded on 6% polyacrylamide gels. After electrophoresis, proteins were transferred to PVDF membranes, which were blocked for 1 h in TBS-Triton X-100/2% milk before the primary antibody was applied overnight at 4°C (1:1000; anti-FANCD2 H-300, Santa Cruz Biotechnology, Heidelberg, Germany; anti-FANCC ab5065, Abcam, Cambridge, UK). HA-tagged FANCC was detected using an anti-HA antibody (1:1000; 12CA5, Santa Cruz). Antibodies directed against anti-RAD50 (1:5000; 13B3, GeneTex, San Antonio, TX), anti-p84 (1:1000; ab487, Abcam), anti-CUL1 (1:1000; 2H4C9, Invitrogen, Karlsruhe, Germany) or anti-β-ACTIN (1:10.000; AC-15, Sigma) served as loading controls. The membranes were washed and stained with anti-rabbit or anti-mouse HRP-conjugated antibodies (1:2000 to 1:10.000; GE Healthcare, Freiburg, Germany). Enhanced chemo-luminescence was elicited using SuperSignal West Pico substrate (Thermo Scientific, Schwerte, Germany) according to the manufacturer's instructions.

### Nuclear focus formation assays

Experiments were performed as described before [74]. In brief, cells were grown on coverslips until ~80% confluency and were exposed to ionizing γ-radiation (IR) at 15 Gy using a cesium-137 irradiator. After incubation for 6 h, the cells were fixed using 3.7% formaldehyde and -20°C methanol. The cells were permeabilized using Triton X-100 and incubated in blocking buffer (PBS +2% bovine serum albumin +0.5% Triton X-100) for 30 min. Consecutively, the cells were labelled using antibodies against FANCD2 (FI-17) or RAD51 (H-92) (Santa Cruz), respectively, for 2 h at room temperature. After washing, Alexa 488 goat anti-mouse or anti-rabbit antibody (Invitrogen) was applied for 1.5 h. Nuclei were counterstained using Hoechst 33342 (Roche Diagnostics, Mannheim, Germany), mounted and analyzed using a fluorescence microscope and Axiovision Software (Carl Zeiss AG; Oberkochen, Germany). The settings were kept identical for all samples.

### Gene complementation studies

For FA complementation group determination, the cell line HuH-7 was transduced with retroviral constructs containing full-length cDNAs of *FANCA*, *FANCB*, *FANCC*, *FANCE*, *FANCF*, *FANCG *or *FANCL *and analyzed for cell cycle arrest upon treatment with MMC and for restoration of FANCD2 monoubiquitination, as previously described [26]. For isogenic studies, HuH-7 cells were stably transduced with either HA-tagged pMSCV-*FANCC *(HuH-7/*FANCC*) or the corresponding pMSCV empty control vector (HuH-7/ev).

### gDNA sequencing

High molecular weight genomic DNA was prepared using a salting-out technique. Amplification of the *FANCC *exons was performed using published primer sets [75]. PCR products were purified using the GFX PCR DNA and Gel Band Purification kit (GE Healthcare). DNA sequencing of PCR products was performed using ABI-PRISM big-dye terminator chemistry on the ABI 310 instrument (Applied Biosystems, Darmstadt, Germany).

### mRNA expression analyses and cDNA sequencing

Total RNA was extracted from surgical HCC tissues or cell lines using the NucleoSpin RNAII-Kit (Macherey-Nagel, Düren, Germany). RNA was reverse-transcribed using SuperScriptII reverse transcriptase (Invitrogen) and cDNA synthesized. Aberrant splicing of *FANCC *was determined by RT-PCR using the following primer pairs: Fwd 5'-ACTGCCCAAACTGCTGAAG-3' and Rev 5'-GTTCAGACGCTAATGATAAAACCA-3' (spanning the first non-coding exon to coding exon 5), Fwd 5'-TTCTGGACAATCAAAACTTAACTCC-3' and Rev 5'-GCTGCTGCTTCTGGACATT-3' (spanning the coding exons 3 to 13), Fwd 5'-GTAGTCTG CCTCTGGCTTCG-3' and Rev 5'-TTGAGGAGAAGGTGCCTGAT-3' (spanning the coding exons 7 to 13). Expression of *FANCC*, *FANCF *and *FANCG *mRNA was validated by RT-PCR as described previously [76]. For sequencing of the complete coding regions of *FANCC*, *FANCF *and *FANCG*, respectively, the corresponding cDNAs were amplified in two (*FANCF*) or three (*FANCC *and *FANCG*) overlapping PCR reactions (primer sets available on request). Amplified products were sequenced using an ABI-Prism 3100-Avant Sequencer (Applied Biosystems) and sequence changes confirmed at the genomic level by gDNA sequencing. Reference genomic and cDNA sequences of the FA genes are available in the Fanconi Anemia Mutation Database [77].

### Cell proliferation assays

The assays were performed over a broad range of concentrations covering 100% to 0% cell survival. 1,500-2,000 cells/well were plated in 96-well plates, allowed to adhere, and treated. Following incubation for 6 d, the cells were washed, lysed in 100 μl H_2_O, and 0.5% Picogreen (Molecular Probes, Invitrogen) was added. Fluorescence was measured (Cytofluor Series 4000, Applied Biosystems) and growth inhibition calculated as compared to the untreated control samples. Three independent experiments were performed per agent, with each data point reflecting triplicate wells. Error bars represent standard error of the mean (SEM) from three experiments.

### Cell cycle analyses

Cells were seeded in 12-well plates and treated in duplicate for 48 h using various MMC concentrations (ranging from 25 to 200 nM) or were left untreated. The cells were fixed, stained with propidium iodide, and the DNA content per cell was measured using flow cytometry (FACSCalibur, Becton Dickinson, Heidelberg, Germany). The data were analyzed using CELLQuest Pro software (Becton Dickinson). Alternatively, unfixed cells were stained with DAPI at a final concentration of 1 μg/ml in a buffer containing 154 mM NaCl, 1 mM CaCl_2_, 0.5 mM MgCl_2_, 0.1 M TRIS, 0.2% BSA, and 0.1% NP40 for 30 min in the dark. Univariate flow histograms were recorded on an analytical, triple-laser equipped flow cytometer (LSRII, Becton Dickinson) using UV excitation of the DAPI dye. The resulting cell cycle distributions reflecting cellular DNA content were quantified using the MPLUS AV software package (Phoenix Flow Systems, San Diego, CA).

### HpaII restriction enzyme methylation analysis

PCR-based HpaII-restriction assays were performed as described previously [23]. Transcriptional silencing through CpG hypermethylation was analyzed using published primer sets for *FANCF *[23] and *FANCC *[69], respectively. Primers used for *FANCG *were 5'-GAGTGCAATGGCACGATG-3' (forward) and 5'-GCATGCTGGGAGTCGTAGTA-3' (reverse). CpGenome universal methylated or unmethylated DNA (Chemicon - Millipore, Schwalbach am Taunus, Germany), respectively, were used as controls.

## List of abbreviations

FA: Fanconi anemia; GI: gastrointestinal, HCC: hepatocellular carcinoma; ICL: interstrand-crosslink; IC50: inhibitory concentration 50%; LOH: loss of heterozygosity; MMC: mitomycin C; SNP: single nucleotide polymorphism; RT-PCR: reverse-transcription PCR; TACE: transarterial chemoembolization

## Competing interests

The authors declare that they have no competing interests.

## Authors' contributions

AP was involved in study design, acquisition, analysis and interpretation of most of the data and drafted the manuscript. KN and DS carried out the complementation studies and parts of the genomic sequencing. UP, AZ, BT and DM performed and interpreted most of the immunoblotting assays. UP and AZ further performed and interpreted drug sensitivity assays and AZ additionally performed parts of the genomic sequencing. AR, EDT and AZ performed and interpreted the FACS analyses. SO performed immunoblotting, immunofluorescence and interpreted the nuclear focus formation assays. BT and FB engineered the stably transduced HuH-7 pMSCV lines. GUD provided and analyzed cholangiocellular carcinoma cell lines. WET provided the HCC tumor specimens. HH provided the retroviral constructs. EDT, CS, BG and DS participated in the conception and the design of the study or provided important intellectual content. EG conceived the project, coordinated the experiments, directed the analysis and interpretation of the data and wrote the final manuscript. All authors read and approved the final manuscript.
